# A Futile Metabolic Cycle of Fatty Acyl Coenzyme A (Acyl-CoA) Hydrolysis and Resynthesis in Corynebacterium glutamicum and Its Disruption Leading to Fatty Acid Production

**DOI:** 10.1128/AEM.02469-20

**Published:** 2021-01-29

**Authors:** Masato Ikeda, Keisuke Takahashi, Tatsunori Ohtake, Ryosuke Imoto, Haruka Kawakami, Mikiro Hayashi, Seiki Takeno

**Affiliations:** a Department of Agricultural and Life Sciences, Faculty of Agriculture, Shinshu University, Nagano, Japan; b Bioprocess Development Center, Kyowa Hakko Bio Co., Ltd., Tsukuba, Ibaraki, Japan; University of Tartu

**Keywords:** *Corynebacterium glutamicum*, mycolic acid-containing bacterium, acyl-CoA thioesterase, acyl-CoA synthetase, futile cycle, fatty acid production

## Abstract

Fatty acyl coenzyme A (acyl-CoA) thioesterase (Tes) and acyl-CoA synthetase (FadD) catalyze opposing reactions between acyl-CoAs and free fatty acids. Within the genome of Corynebacterium glutamicum, several candidate genes for each enzyme are present, although their functions remain unknown. Modified expression of the candidate genes in the fatty acid producer WTΔfasR led to identification of one *tes* gene (*tesA*) and two *fadD* genes (*fadD5* and *fadD15*), which functioned positively and negatively in fatty acid production, respectively. Genetic analysis showed that *fadD5* and *fadD15* are responsible for utilization of exogenous fatty acids and that *tesA* plays a role in supplying fatty acids for synthesis of the outer layer components mycolic acids. Enzyme assays and expression analysis revealed that *tesA*, *fadD5*, and *fadD15* were coexpressed to create a cyclic route between acyl-CoAs and fatty acids. When *fadD5* or *fadD15* was disrupted in wild-type C. glutamicum, both disruptants excreted fatty acids during growth. Double disruption of these genes resulted in a synergistic increase in production. Additional disruption of *tesA* revealed a canceling effect on production. These results indicate that the FadDs normally shunt the surplus of TesA-generated fatty acids back to acyl-CoAs for lipid biosynthesis and that interception of this shunt provokes cells to overproduce fatty acids. When this strategy was applied to a high-fatty-acid producer, the resulting *fadD*-disrupted and *tesA*-amplified strain exhibited a 72% yield increase relative to its parent and produced fatty acids, which consisted mainly of oleic acid, palmitic acid, and stearic acid, on the gram scale per liter from 1% glucose.

**IMPORTANCE** The industrial amino acid producer Corynebacterium glutamicum has evolved into a potential workhorse for fatty acid production. In this organism, we obtained evidence showing the presence of a unique mechanism of lipid homeostasis, namely, formation of a futile cycle of acyl-CoA hydrolysis and resynthesis mediated by acyl-CoA thioesterase (Tes) and acyl-CoA synthetase (FadD), respectively. The biological role of the coupling of Tes and FadD would be to supply free fatty acids for synthesis of the outer layer components mycolic acids and to recycle their excess to acyl-CoAs for membrane lipid synthesis. We further demonstrated that engineering of the cycle in a high-fatty-acid producer led to dramatically improved production, which provides a useful engineering strategy for fatty acid production in this industrially important microorganism.

## Abstract (precis)

The industrial amino acid producer Corynebacterium glutamicum has evolved into a potential workhorse for fatty acid production. In this organism, we obtained evidence showing the presence of a unique mechanism of lipid homeostasis, namely, formation of a futile cycle of acyl-CoA hydrolysis and resynthesis mediated by acyl-CoA thioesterase (Tes) and acyl-CoA synthetase (FadD), respectively.

## INTRODUCTION

Microbial production of fatty acids, lipids, and their related compounds has received significant attention as a renewable source of biofuels and functional nutrients (1). The fermentative processes have been developed using oleaginous fungi, yeasts, and algae (2–4). However, attempts to use naturally nonoleaginous bacteria for that purpose have been increasing in recent years (5–7). For example, it has been demonstrated with Escherichia coli that cytosolic expression of the periplasmic enzyme acyl-acyl carrier protein (acyl-ACP) thioesterase I (TesA) in cells deficient in the β-oxidative fatty acid degradation pathway resulted in the extracellular production of free fatty acids (5), which has recently become a common strategy for fatty acid production by E. coli (8).

**FIG 1:**
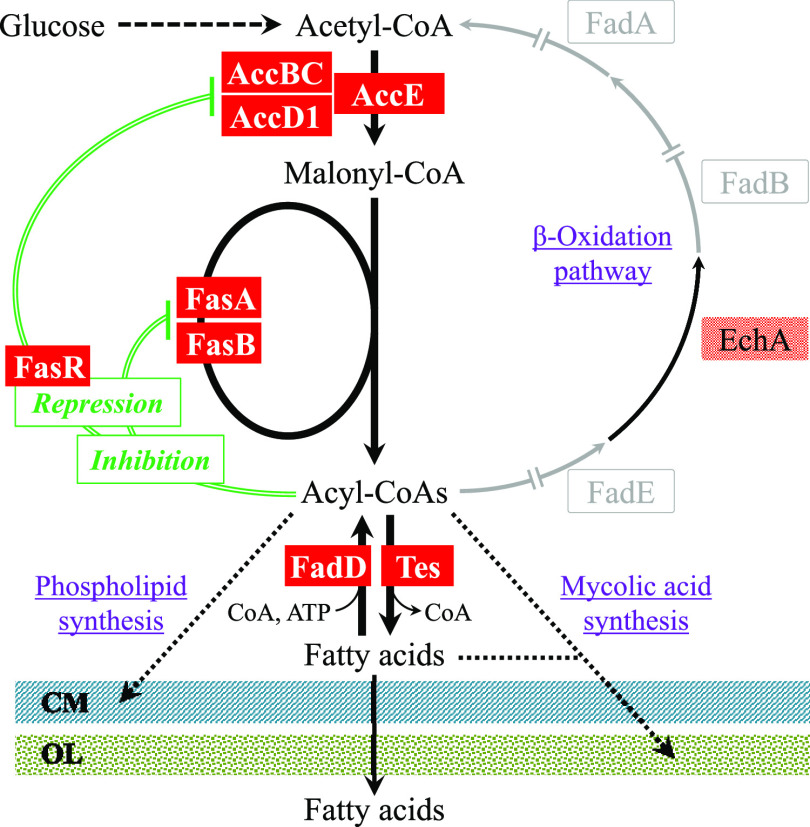
Lipid metabolism and its predicted regulatory mechanism in C. glutamicum. For fatty acid biosynthesis, C. glutamicum uses two type I fatty acid synthases (FAS-I), FasA and FasB, in addition to acetyl-CoA carboxylase (Acc), consisting of AccBC, AccD1, and AccE. The products of the FAS-I pathway are believed to be acyl-CoAs (51), which are used for the synthesis of membrane phospholipids and the outer layer components mycolic acids. FasB is also involved in the synthesis of octanoic acid (C_8_), a precursor of lipoic acid (12). This organism is naturally unable to degrade fatty acids, since three genes (gray arrows) responsible for the β-oxidation of fatty acids are missing from the C. glutamicum genome (18). Based on knowledge of related bacteria, acyl-CoAs are thought to inhibit Acc, FasA, and FasB (52, 53). The repressor protein FasR, combined with the effector acyl-CoAs, represses the expressions for *accD1*, *fasA*, and *fasB* (19, 54). Repression and predicted inhibition are indicated by double lines. Arrows with solid and dotted lines represent single and multiple enzymatic processes, respectively. FadE, acyl-CoA dehydrogenase; EchA, enoyl-CoA hydratase; FadB, hydroxyacyl-CoA dehydrogenase; FadA, ketoacyl-CoA reductase; CM, cytoplasmic membrane; OL, outer layer.

Our group and others have developed the amino acid-producing microorganism Corynebacterium glutamicum as another platform for the production of fatty acids and their related compounds (9–14). With respect to fatty acid metabolism, this organism has at least three inherent properties that are distinct from those of E. coli (Fig. 1): (i) the presence of a eukaryotic multifunctional type I fatty acid synthase (FAS-I) system comprising FasA and FasB (15, 16), in contrast to E. coli, which employs an individual nonaggregating type II fatty acid synthase (FAS-II) system (17); (ii) a lack of the β-oxidation pathway involving fatty acid degradation (10, 18); and (iii) a high level of cytoplasmic acyl coenzyme A (acyl-CoA) thioesterase (Tes) activity (10). Owing to these unique C. glutamicum properties, only a loss-of-function mutation of *fasR*, which encodes a fatty acid biosynthesis repressor protein (19), gives rise to fatty acid production without modification to the Tes enzyme (10). This, however, raises the question of why this organism has naturally high cytoplasmic Tes activity, in contrast to E. coli. C. glutamicum is likely to possess a unique mechanism that differs from that of E. coli for maintaining lipid homeostasis. This prompted us to focus on Tes and, at the same time, its opposing enzyme acyl-CoA synthetase (FadD), which catalyzes the activation of free fatty acids to acyl-CoAs using ATP (Fig. 1).

With respect to the corresponding two enzymes in E. coli, considerable information is available regarding the enzymatic and functional properties as well as the genes. For Tes, three isoforms, Tes I, II, and III, have so far been identified (20, 21). Tes I, encoded by *tesA*, is specific for C_12_ to C_18_ acyl-thioesters, while it is unlikely to have access to acyl-thioesters, because native Tes I is a periplasmic enzyme (22). Thus, the physiological role of this enzyme remains unknown. A second Tes II, a cytosolic enzyme encoded by *tesB*, has broader substrate specificity and is active for C_6_ to C_18_ acyl-thioesters. However, since no obvious physiological or biochemical defect was observed in E. coli with *tesB* overexpression or deletion (23, 24), the exact physiological function of Tes II in lipid metabolism has not been established. A third Tes III, which is encoded by *fadM* (*ybaW*), has been shown to be a long-chain acyl-CoA thioesterase that is most active with 3,5-tetradecadienolyl-CoA, a minor metabolite of the β-oxidation of oleic acid (21). Although the *fadM* gene is not essential for growth, it has been demonstrated to be inductively expressed when E. coli is grown on oleic acid as the sole carbon source (21, 25). Based on these findings, Tes III has been assumed to perform a backup role in fatty acid β-oxidation.

On the other hand, E. coli FadD is a single enzyme encoded by *fadD* and has a broad substrate specificity for fatty acids of medium to long chain lengths (26), although E. coli has another enzyme, FadK, that is involved in the activation of short-chain fatty acids solely under anaerobic conditions (27). The *fadD* gene has been reported to be derepressed after cells enter the stationary phase of growth, where free fatty acids are generated from the degradation of membrane lipids (28). Thus, the physiological role of E. coli FadD is to activate fatty acids that originated from membrane lipids to generate acyl-CoAs, which are further metabolized by the β-oxidation pathway to generate a source of carbon and energy (28).

The C. glutamicum genome indicates the presence of two and five putative genes for Tes and FadD, respectively, although the functions of most remain unclear. In this study, we aimed to (i) identify the *tes* and *fadD* genes involved in fatty acid production by C. glutamicum and (ii) clarify the roles of each gene in fatty acid metabolism and production. Here, we obtained evidence showing that the relevant Tes and FadD enzymes form a futile metabolic cycle of acyl-CoA hydrolysis and resynthesis during growth on glucose, providing a new mechanism for maintaining both lipid homeostasis and fatty acid production. Why does this organism need to employ such a unique futile cycle? Its physiological significance is discussed below.

## RESULTS

### *In silico* analysis of candidate genes for FadD and Tes.

The C. glutamicum genomic database (GenBank accession number BA000036) indicates the presence of the following five genes encoding putative FadD proteins (see Fig. S1A in the supplemental material): *fadD1* (Cgl0284, NCgl0279), *fadD5* (Cgl0400, NCgl0388), *fadD4* (Cgl1198, NCgl1151), *fadD15* (Cgl2296, NCgl2216), and *fadD32* (Cgl2872, NCgl2774). All of these genes contain ATP/AMP and fatty acyl-CoA synthetase (FACS) motifs homologous to those in E. coli FadD (Fig. S1B) (29). To date, however, there have been no reports on their functions, except for *fadD32*, which is located in a chromosomal cluster with *accD3* and *pks* (Fig. S1A) and has been assumed to be involved in the synthesis of mycolic acids by activating free fatty acids to form acyl-AMP (14, 30). On the other hand, C. glutamicum possesses two annotated *tes* genes, *tesB* (Cgl1664, NCgl1600) and *tesA* (Cgl2451, NCgl2365), whose functions remain to be clarified (Fig. S2A). The deduced amino acid sequence of the *tesA* product, unlike that of the *tesB* product, has an active-site motif that is homologous to the E. coli *ybaW* product Tes III, a long-chain acyl-CoA thioesterase (Fig. S2B) (21), suggesting the involvement of *tesA* in the hydrolysis of long-chain fatty acyl-CoAs.

### *In vivo* identification of the genes responsible for the conversion between long-chain fatty acids and their CoA derivatives.

Our first task was to identify the *fadD* and *tes* genes responsible for the transfer and release, respectively, of CoA between long-chain fatty acids and their CoA derivatives. Based on the catalytic reactions, it was reasonable to expect that the intended *fadD* gene negatively affects fatty acid production when amplified in a fatty acid producer while the intended *tes* gene plays a pivotal role in fatty acid production. Initially, to identify the types of *fadD* genes present among the five candidate *fadD* genes, their coding regions were individually cloned on a multicopy vector so as to be constitutively expressed under the control of the promoter of the endogenous *gapA* gene, encoding glyceraldehyde 3-phosphate dehydrogenase, to generate plasmids pCfadD1, pCfadD4, pCfadD5, pCfadD15, and pCfadD32. Each plasmid was introduced into the C. glutamicum fatty acid-producing strain WTΔfasR, and the resulting plasmid carriers were compared with the control vector carrier for fatty acid production when cultivated in minimal medium (MM) (1% glucose). As shown in Table 1, plasmids pCfadD5 and pCfadD15 brought about approximately 25% and 28% decreases in fatty acid production, respectively, while the other three plasmids had only marginal or relatively small effects on production. These data suggest that *fadD5* and *fadD15* play significant roles in getting free fatty acids back to their CoA derivatives. Although the pCfadD32 carrier showed a 15% decrease in production, we believed that this was probably due to the redirection of carbon into mycolic acid synthesis, considering the predicted role of FadD32, namely, activation of free fatty acids to form acyl-AMP, a precursor for mycolic acid synthesis (14, 30).

**TABLE 1:** Effect of modified *fadD* or *tesA* expression on fatty acid production by strain WTΔfasR

Strain (plasmid)	Carbon source	Growth (OD_660_)	Fatty acid concn (mg/liter)^*a*^			
			Oleic acid	Palmitic acid	Stearic acid	Total
WTΔfasR (vector)	Glucose	8.2 ± 0.4	91.4 ± 3.3	73.0 ± 2.5	7.6 ± 0.6	172.0 ± 6.3 (1.0)
WTΔfasR (pCfadD1)	Glucose	8.0 ± 0.3	83.7 ± 2.2	71.3 ± 4.3	3.0 ± 0.8	157.9 ± 7.1 (0.92)
WTΔfasR (pCfadD4)	Glucose	7.8 ± 0.3	89.4 ± 1.7	72.2 ± 0.9	9.5 ± 0.4	171.2 ± 2.9 (1.0)
WTΔfasR (pCfadD5)	Glucose	8.1 ± 0.6	64.5 ± 6.7	57.1 ± 4.4	7.4 ± 1.0	129.1 ± 11.9 (0.75)
WTΔfasR (pCfadD15)	Glucose	8.0 ± 0.7	66.3 ± 6.8	50.5 ± 4.7	7.1 ± 1.6	124.1 ± 13.2 (0.72)
WTΔfasR (pCfadD32)	Glucose	8.2 ± 0.6	74.2 ± 5.0	63.2 ± 4.8	7.6 ± 1.9	145.5 ± 11.5 (0.85)
WTΔfasRΔtesA	Glucose	5.2 ± 0.3	2.3 ± 0.3	1.6 ± 0.2	0.2 ± 0.04	4.1 ± 0.4 (0.02)
WTΔfasRΔtesA (pCtesA)	Glucose	8.1 ± 0.5	107.2 ± 7.1	78.6 ± 3.1	9.7 ± 0.7	195.5 ± 10.6 (1.14)
WTΔfasR	Glucose	8.6 ± 0.4	90.2 ± 4.5	78.7 ± 3.1	6.7 ± 0.4	175.6 ± 8.0 (1.02)
	*myo*-Inositol	8.0 ± 0.5	97.3 ± 5.1	64.6 ± 3.4	7.7 ± 0.7	169.6 ± 8.9 (0.99)
WTΔfasRtesA^iol^	Glucose	8.5 ± 0.4	3.6 ± 0.2	2.1 ± 0.2	0.3 ± 0.03	6.0 ± 0.4 (0.03)
	*myo*-Inositol	8.3 ± 0.6	94.4 ± 3.9	69.7 ± 2.7	4.7 ± 0.5	168.8 ± 5.3 (0.98)

a Production was carried out using 300-ml baffled Erlenmeyer flasks containing 30 ml of MM (1% glucose or 1% *myo*-inositol). After glucose or *myo*-inositol was consumed, total lipids, including free fatty acids, were extracted from the culture supernatant to determine free fatty acids by gas chromatography. Value are means and standard deviations of the results from three independent experiments. Values in parentheses are relative to the titer obtained with the control strain, WTΔfasR (vector).

As for *tes*, the above-mentioned *in silico* analysis suggested that the gene annotated as *tesA* is a more likely candidate for long-chain fatty acyl-CoA hydrolysis than the other *tesB* gene. If so, the loss of *tesA* function should impair the ability of strain WTΔfasR to produce fatty acids. In fact, disruption of *tesA* resulted in an almost complete loss of fatty acid production ability, and this phenotype was fully complemented by the plasmid-mediated expression of *tesA* (Table 1). However, since the *tesA*-disrupted strain WTΔfasRΔtesA showed an approximately 40% lower growth level than its parent, WTΔfasR, as can be seen from the optical density (OD) (Table 1), there remained a possibility that the reduced growth might affect fatty acid production. To avoid this, we expressed the chromosomal *tesA* gene under the control of the *myo*-inositol-inducible promoter of *iolT1*, encoding a *myo*-inositol transporter in strain WTΔfasR, followed by examination for fatty acid production under the conditions with glucose and with *myo*-inositol. The resulting strain, WTΔfasRtesA^iol^, grew well regardless of the carbon sources and exhibited a *myo*-inositol-dependent fatty acid-producing phenotype, while the control WTΔfasR strain produced fatty acids under both conditions (Table 1). These data strongly suggest that *tesA* is responsible for long-chain fatty acyl-CoA hydrolysis and is thus essential for fatty acid production.

### Significance of *fadD5* and *fadD15* in oleic acid utilization.

**FIG 2:**
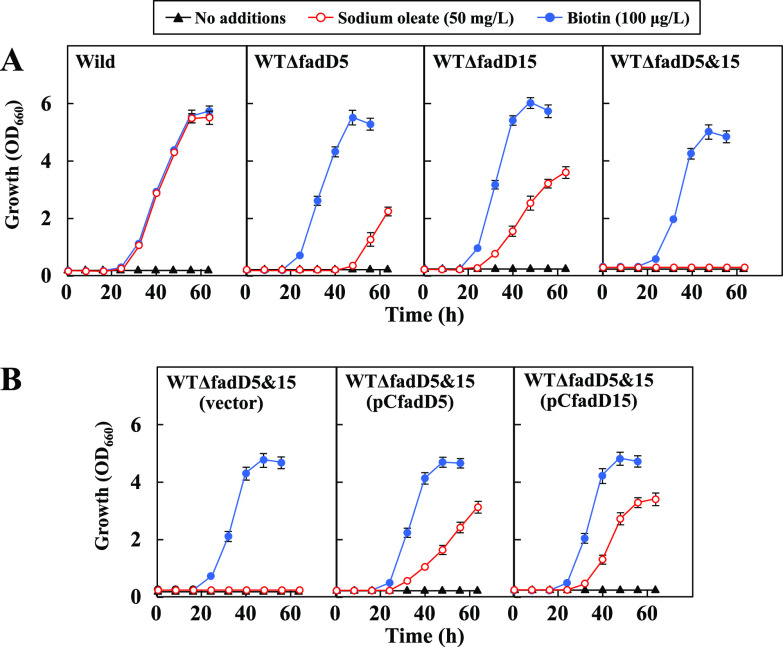
Effect of disrupted *fadD* genes on oleic acid utilization (A) and effect of *fadD5* or *fadD15* expression on oleic acid utilization by the double disruptant WTΔfadD5&15 (B). Cultivations were carried out in biotin-free MM with no additions or 50 mg/liter sodium oleate. For reference, the growth profiles in biotin (100 μg/liter)-supplemented MM are shown as controls. Values are means and standard deviations of the results from three independent cultures.

Naturally biotin-auxotrophic C. glutamicum can grow on glucose even under biotin-free conditions, provided that oleic acid is used to supplement the medium (31). This is because exogenous oleic acid is activated to oleoyl-CoA by FadD and is then utilized for membrane lipid biosynthesis. Accordingly, based on the above-mentioned results, it is reasonable to expect that the loss of *fadD5* and/or *fadD15* functions would result in impaired growth even in the presence of oleic acid under biotin-free conditions. To verify this, we constructed *fadD5*- and *fadD15*-disrupted strains, designated WTΔfadD5 and WTΔfadD15, respectively, and their double disruptant, designated WTΔfadD5&15, from the wild-type strain. As shown in Fig. 2A, the wild-type strain (WT) grew normally under biotin-free and oleate-supplemented conditions, while strains WTΔfadD5 and WTΔfadD15 exhibited the expected phenotypes of retarded growth under the same culture conditions. In the case of strain WTΔfadD5&15, no growth was observed, although the presence of biotin in the culture restored the growth. We further confirmed that the inability of strain WTΔfadD5&15 to utilize exogenous oleate was complemented, although only partially, by the plasmid-mediated expression of *fadD5* or *fadD15* (Fig. 2B). These results fortify our earlier conclusion that *fadD5* and *fadD15* play a role in converting long-chain fatty acids to their CoA derivatives.

### Significance of the *tesA* gene in growth.

**FIG 3:**
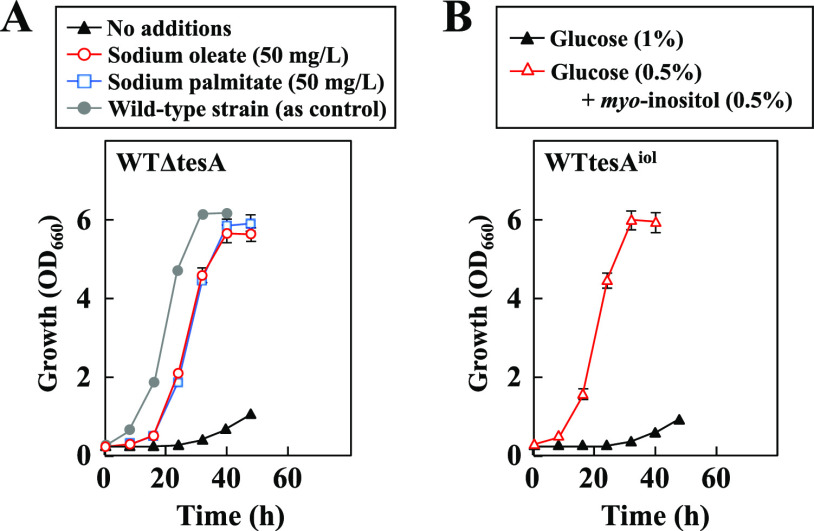
Growth characteristics of the *tesA* disruptant WTΔtesA and the *myo*-inositol-dependent *tesA*-expressing strain WTtesA^iol^. (A) Strain WTΔtesA was cultivated in MM with no additions, 50 mg/liter sodium oleate, or 50 mg/liter sodium palmitate. For reference, the growth profile of the wild-type strain in MM is shown as a control. (B) Strain WTtesA^iol^ was cultivated in MM containing 1% glucose or 0.5% glucose plus 0.5% *myo*-inositol. Values are means and standard deviations of the results from three independent cultures.

Although the data discussed above indicate the essential role of the genomic *tesA* gene in fatty acid production, its physiological function remains to be clarified. Thus, we examined the phenotype of deficiency in *tesA* under the wild-type background. As shown in Fig. 3A, the *tesA*-disrupted strain WTΔtesA showed impaired growth on glucose, and this phenotype was almost fully recovered under the conditions of supplementation with either oleate or palmitate. To further confirm this, we expressed chromosomal *tesA* under the control of the *myo*-inositol-inducible promoter of *iolT1*. When the resulting strain, WTtesA^iol^, was cultivated on 1% glucose or 0.5% glucose plus 0.5% *myo*-inositol, it exhibited *myo*-inositol-dependent growth (Fig. 3B). These results indicate that the C. glutamicum wild type requires the *tesA* function for normal growth on glucose and its deficiency causes the requirement for the free fatty acid oleate or palmitate.

### Enzymatic activities and transcript levels in glucose-grown cells.

The results so far described suggest that *fadD5*, *fadD15*, and *tesA* are all expressed in wild-type cells grown on glucose. However, if TesA and FadDs operate simultaneously, a futile cycle that leads to the hydrolysis of ATP as the net effect would result (Fig. 1), which would be unreasonable. Therefore, we examined whether the coexpression actually occurred in wild-type cells by measuring the enzymatic activities and transcript levels. Initially, we determined the enzymatic activities using soluble fractions prepared from cells in the late exponential phase of growth on glucose. As for FadD, although significant activity was detected in wild-type cells (11.0 ± 0.4 mU/mg of protein), double disruption of *fadD5* and *fadD15* resulted in activity being reduced to a marginal level (1.6 ± 0.9 mU/mg). The *fadD5*- and *fadD15*-disrupted strains showed moderate levels of activity (5.5 ± 0.3 mU/mg and 7.1 ± 0.4 mU/mg, respectively). On the other hand, a relatively high level of Tes activity was detected in wild-type cells (276 ± 53 mU/mg), in contrast to a trace level in the *tesA*-disrupted strain.

Next, we investigated the transcript levels of *fadD5*, *fadD15*, and *tesA* in the wild-type strain and its derived *fadD5*-, *fadD15*-, *fadD5*- and *fadD15*-, and *tesA*-disrupted strains during growth on glucose (Fig. S3). The data for the three genes in each disruptant are presented as relative to values obtained for the corresponding genes in the wild-type strain. The transcript level of each gene was shown to fall to a negligible level as a consequence of the disruption of the corresponding gene, indicating that the three genes are all expressed in wild-type cells grown on glucose.

This series of data not only reconfirmed our earlier conclusion that the intended Tes and FadD activities are specified by *tesA* and the two *fadD* genes (*fadD5* and *fadD15*), respectively, but also verified that the two opposing reactions operate simultaneously in wild-type cells during growth on glucose.

### Effects of *fadD5* and *fadD15* disruption on fatty acid production.

**FIG 4:**
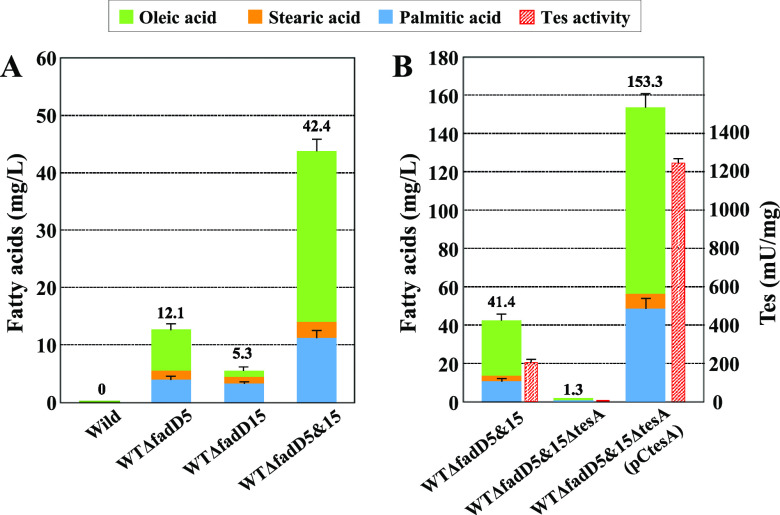
Fatty acid production by *fadD* disruptants (A) and the double disruptant WTΔfadD5&15 with disrupted and amplified *tesA* (B). All cultivations were carried out in 30 ml of MM (1% glucose) in 300-ml baffled Erlenmeyer flasks. After glucose was consumed, total lipids, including free fatty acids, were extracted from the culture supernatant to determine levels of free fatty acids by gas chromatography. Specific activities of Tes in strains WTΔfadD5&15 with disrupted and amplified *tesA* are also shown (B). Values are means and standard deviations of the results from three independent experiments.

The findings that *tesA* and the two *fadD* genes were coexpressed in wild-type cells during growth on glucose suggest the formation of a cyclic metabolic route between long-chain fatty acids and their CoA derivatives. If so, and considering that this organism naturally lacks the β-oxidation pathway involving fatty acid degradation, it is likely that blockage of the cycle at the FadD step would provoke cells to accumulate fatty acids which are normally synthesized by this organism, namely, oleic acid, palmitic acid, and stearic acid. Furthermore, the potential to accumulate the fatty acids should be cancelled by the additional disruption of *tesA.* Based on these assumptions, we examined whether the disruption of *fadD5* and/or *fadD15* causes fatty acid production in wild-type cells during cultivation in MM with 1% glucose. As shown in Fig. 4A, significant amounts of fatty acids were found to accumulate in the cultures of the *fadD5*-disrupted and *fadD15*-disrupted strains WTΔfadD5 and WTΔfadD15, respectively, whereas the control wild type produced no detectable fatty acids. When both *fadD5* and *fadD15* were disrupted in wild-type cells, the resulting double disruptant, WTΔfadD5&15, exhibited synergistically increased production under the same conditions.

Next, we examined whether *tesA* played a key role in fatty acid production by strain WTΔfadD5&15. As shown in Fig. 4B, the disruption of *tesA* in strain WTΔfadD5&15 led to an almost complete loss of fatty acid production ability concomitantly with a loss of Tes activity, while plasmid-mediated amplification of *tesA* in the triple disruptant WTΔfadD5&15ΔtesA resulted in dramatically increased production with increased Tes activity. It is noteworthy that when *tesA* was amplified in the wild-type strain, fatty acid production was not observed (data not shown).

**FIG 5:**
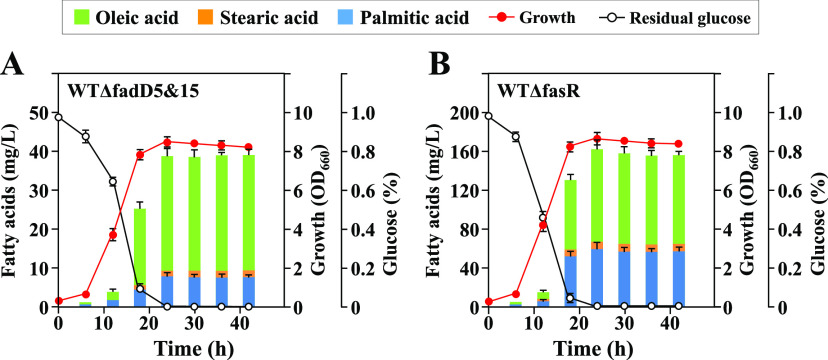
Fermentation profiles by strains WTΔfadD5&15 (A) and WTΔfasR (B). Two different lines of fatty acid producers, WTΔfadD5&15 and WTΔfasR, were cultivated in 300-ml baffled Erlenmeyer flasks containing 30 ml of MM (1% glucose). Levels of free fatty acids, as well as growth and residual glucose, were measured every 6 h. Values are means and standard deviations of the results from three independent experiments.

To further confirm that fatty acid production by strain WTΔfadD5&15 occurred during growth on glucose and not after cells entered the stationary phase, we examined its fermentation profile using the typical fatty acid producer WTΔfasR as a control. As shown in Fig. 5A, fatty acid production by strain WTΔfadD5&15 occurred mainly in the late exponential phase of growth on glucose and was stopped after glucose was consumed, which was almost the same profile as that for the control strain WTΔfasR (Fig. 5B).

These results reinforce our conclusions that TesA and the two FadDs normally form a cycle between long-chain fatty acids and their CoA derivatives (Fig. 1) and that overproduction of fatty acids results when the cycle is blocked at the FadD step.

### Engineering a high-fatty-acid producer through disrupted *fadD* genes and amplified *tesA*.

**FIG 6:**
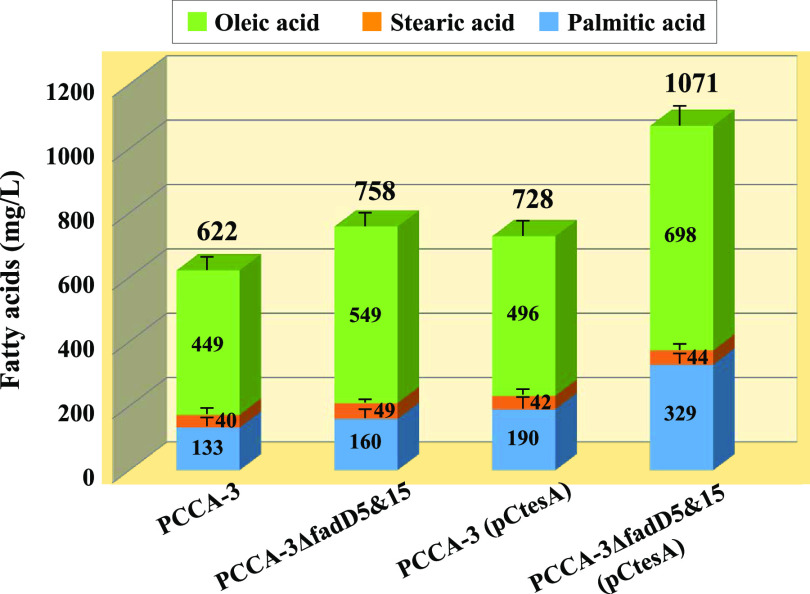
Fatty acid production by strain PCCA-3 with disrupted *fadD* genes and/or amplified *tesA.* Production was carried out using 300-ml baffled Erlenmeyer flasks containing 30 ml of MM (1% glucose). After glucose was consumed, total lipids were extracted from the culture broth containing cells to determine free fatty acids by gas chromatography. Values are means and standard deviations of the results from three independent cultures.

As mentioned above, engineering of the metabolic cycle consisting of TesA and FadDs, specifically, a combination of disrupted *fadD*5 and *fadD15* and amplified *tesA* (referred to as the TesFad method), allows C. glutamicum to produce a significant amount of fatty acids, even on a wild-type background (Fig. 4B). The TesFad method differs in production mechanism from the general method, namely, deregulation of fatty acid synthesis, and is expected to be a new strategy for fatty acid production in C. glutamicum. To substantiate the method, we applied it to our best fatty acid producer, PCCA-3 (14). This producer was developed by assembling four positive mutations (*fasR20*, *fasA63*^up^, *fasA2623*, and *accD3*^A433T^) in the wild-type genome, and it has the capability of producing approximately 600 mg/liter of fatty acids, which consist mainly of oleic acid (449 mg/liter), palmitic acid (133 mg/liter), and stearic acid (40 mg/liter), in flask cultivation with 1% glucose (Fig. 6). Using this strain as a host, we examined the effects of the TesFad method on fatty acid production. As shown in Fig. 6, disruption of the two *fadD* genes alone (designated strain PCCA-3ΔfadD5&15) or plasmid-mediated amplification of *tesA* alone [designated strain PCCA-3(pCtesA)] had relatively small effects on production, but their combination [resulting in strain PCCA-3ΔfadD5&15(pCtesA)] resulted in significantly increased production that achieved a titer of 1,071 mg/liter with a conversion yield of approximately 10% on glucose. These data demonstrate that the effects of the TesFad method are not offset by the performance that strain PCCA-3 has already acquired, and the method could therefore be a useful addition to strain improvement for fatty acid production in this organism. It should be noted that, despite a large increase in the total amount of fatty acids, the fatty acid composition of oleic acid, palmitic acid, and stearic acid remained substantially unchanged when the TesFad method was applied (Fig. 6). It is also noteworthy that no significant by-production of glutamic acid was observed in the engineered strain, suggesting that the engineering would not affect the cell surface structure enough to elicit glutamic acid production.

## DISCUSSION

In this study, we identified Cgl2451 (*tesA*) as the *tes* gene responsible for the hydrolysis of long-chain fatty acyl-CoAs into free fatty acids, as well as two *fadD* genes, Cgl0400 (*fadD5*) and Cgl2296 (*fadD15*), that mediate its opposing reaction in C. glutamicum. Interestingly, disruption of the two *fadD* genes allowed wild-type C. glutamicum to produce considerable amounts of fatty acids (a mixture of oleic acid, palmitic acid, and stearic acid) during growth on glucose. Since this effect was mostly cancelled by the simultaneous disruption of *tesA*, the secreted fatty acids are assumed to be generated from acyl-CoAs in a *tesA*-dependent manner. These results indicate that TesA and the two FadDs normally form a cyclic metabolic route between acyl-CoAs and long-chain fatty acids in this organism (Fig. 1), thus provoking cells to overproduce fatty acids, particularly in situations when the cycle is intercepted at the FadD step. Fatty acid production based on this new metabolic cycle has not yet been reported in the literature.

**FIG 7:**
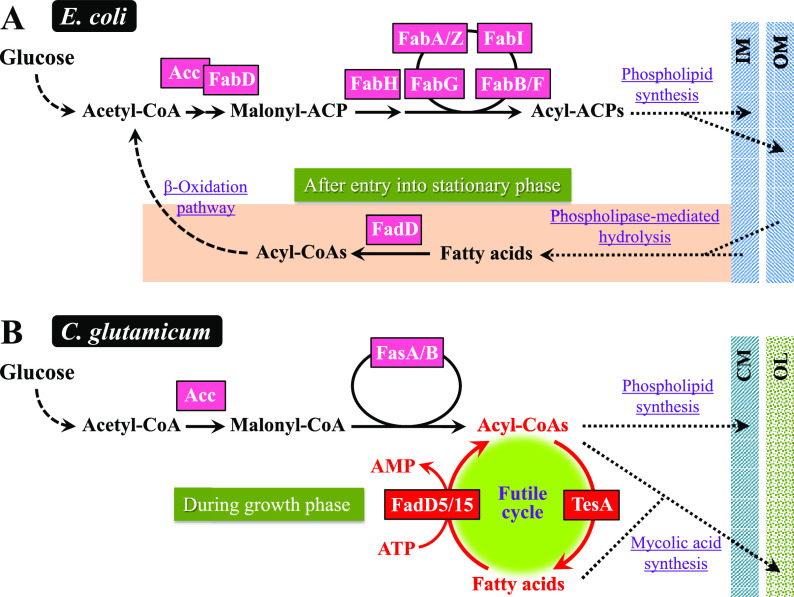
Proposed lipid metabolism in E. coli (A) and C. glutamicum (B). (A) In the case of E. coli, cells grown on carbon sources usually synthesize acyl-acyl carrier proteins (acyl-ACPs) to build membrane lipids, irrespective of the functions of Tes and FadD. After entry into the stationary growth phase, membrane lipids of cells are subject to phospholipase-mediated hydrolysis to release free fatty acids, which are then activated to acyl-CoAs by FadD and subsequently metabolized by the β-oxidation pathway to generate a source of carbon and energy. In the case of *fadD* mutants of E. coli, the fatty acids cannot be activated to acyl-CoAs and are thus destined to be excreted extracellularly. (B) In contrast, C. glutamicum needs to hydrolyze some portion of acyl-CoAs by TesA to supply free fatty acids for the synthesis of the outer layer of mycolic acids. In this context, a surplus of the TesA-generated free fatty acids is recycled to acyl-CoAs by the two FadDs (FadD5/15) for phospholipid synthesis. Therefore, interception of the cyclic metabolic route at the FadD step results in fatty acid overproduction. Although concomitant activities of acyl-CoA hydrolysis and resynthesis create a futile cycle, this has significance as the recycling system of excess fatty acids, especially in an organism that cannot use free fatty acids as a source of carbon and energy. IM, inner membrane; OM, outer membrane; CM, cytoplasmic membrane; OL, outer layer.

In certain other bacteria, such as E. coli and the nitrogen-fixing bacterium Sinorhizobium meliloti, *fadD* mutants accumulate free fatty acids after entering the stationary phase of growth (32). This phenomenon has been explained as follows. Cells grown on carbon sources, such as sugars, usually synthesize acyl-thioesters to build membrane lipids, irrespective of the functions of Tes and FadD. Once the carbon sources are used up, the membrane lipids of cells are subject to phospholipase-mediated hydrolysis to release free fatty acids, which are then activated to acyl-CoAs by FadD and subsequently metabolized by the β-oxidation pathway to generate a source of carbon and energy (Fig. 7A). In fact, the *fadD* gene of E. coli has been reported to be inductively expressed after cells enter the stationary growth phase (28). In the case of the *fadD* mutants of those bacteria, fatty acids released from membrane lipids in the stationary phase cannot be activated to acyl-CoAs scheduled for β-oxidation and are thus destined to be excreted extracellularly. This mechanism differs from that of C. glutamicum, because in E. coli and S. meliloti, free fatty acids are released from membrane lipids in the stationary phase independently of Tes, whereas in C. glutamicum, fatty acids are Tes-dependently generated from acyl-CoAs during growth on glucose, as depicted in Fig. 7B. The phenomenon of the release of fatty acids has also been reported for a Saccharomyces cerevisiae mutant deficient in its respective system of fatty acid activation (33–35). In this case, it was also established that the accumulated free fatty acids originate from phospholipids (34) or lipid droplets (35) as a consequence of lipid turnover processes.

In the culture of the C. glutamicum *fadD* mutant WTΔfadD5&15, the accumulation of fatty acids started in the early growth phase and stopped when glucose was used up. This profile means that wild-type C. glutamicum operates TesA during growth on glucose to generate free fatty acids, which are usually recycled back to acyl-CoAs by FadD5 and FadD15. This hypothesis is supported by the copresence of Tes and FadD activities in glucose-growing wild-type cells. However, the question of why the organism needs to operate TesA during growth on glucose remains. This appears to be related to a unique feature of its cell envelope. This organism has an outer layer of mycolic acids that are synthesized from free long-chain fatty acids as precursors (30). Therefore, some portion of acyl-CoAs need to be hydrolyzed by TesA to supply free fatty acids for mycolic acid synthesis (Fig. 7B). In this context, a surplus of the TesA-generated free fatty acids would be recycled to acyl-CoAs by the FadDs for biosynthesis of membrane lipids. Taken together, the biological role of the coupling of TesA and FadDs would be to supply free fatty acids for the synthesis of the outer layer of mycolic acids and also to recover their excess (Fig. 7B). The free fatty acids observed in our study are the consequence of interrupted fatty acid recycling.

The TesA-FadD cycle that we proposed for C. glutamicum causes the hydrolysis of ATP as the net effect and thus creates a so-called futile cycle. If this is correct, a question arises as to why this organism employs such an energy-wasting cycle in lipid metabolism. It is likely that the best way for this organism to reconcile satisfying the need for free fatty acids for mycolic acid synthesis with saving energy would be to make the TesA enzyme feedback sensitive to free fatty acids. Nevertheless, to the best of our knowledge, no regulatory mechanism such as this has been reported for any Tes enzyme. Against this backdrop, this organism has had no choice but to develop an alternative method. Considering that C. glutamicum naturally lacks the β-oxidative fatty acid degradation pathway, it is most wasteful for this organism to accumulate a surplus of the TesA-generated free fatty acids. Thus, C. glutamicum might have developed the TesA-FadD cycle as a less energy-wasting and more rational system.

In this study, we demonstrated with our C. glutamicum fatty acid producer that interception of the TesA-FadD futile cycle at the FadD step and the concomitant increase in Tes activity led to dramatically improved production (Fig. 6). The conversion yield on glucose exceeded 10% (wt/wt), which is comparable to the yields reported for the typical fatty acid producers of E. coli (7, 8). This indicates not only the usefulness of this engineering strategy but also the potential of this industrial microorganism as a fatty acid producer. As described in the introduction, previous attempts to produce fatty acids using nonoleaginous bacteria, including E. coli and C. glutamicum, have aimed mainly at the deregulation of fatty acid biosynthesis. In contrast, the engineering strategy presented here focuses on the new mechanism of lipid homeostasis found in C. glutamicum. Specifically, the concept of this engineering strategy is not the deregulation of fatty acid biosynthesis but the blockage of the fatty acid-recycling system that is intrinsic to this organism. Medium-chain fatty acids and dicarboxylic acids have also attracted much attention recently (8) and are potential targets for production by this organism. Although much remains to be clarified in regard to the functions of *fadD1*, *fadD4*, and *tesB*, their products are expected to be involved in the synthesis of those compounds, and our future research will examine this further.

## MATERIALS AND METHODS

### Bacterial strains.

All strains used in this study are derivatives of the C. glutamicum wild-type strain ATCC 13032. The fatty acid producer WTΔfasR (10) was derived from ATCC 13032 through the in-frame deletion of *fasR*, which encodes a fatty acid biosynthesis repressor protein. The high-fatty-acid producer PCCA-3 (14) was developed by the so-called “genome breeding” approach (36), in which four specific mutations (*fasR20*, *fasA63*^up^, *fasA2623*, and *accD3*^A433T^) were assembled in the ATCC 13032 genome. The former three mutations contribute to the deregulation of fatty acid biosynthesis, and the latter one is assumed to impair the function of the gene product AccD3 to diminish mycolic acid biosynthesis. E. coli DH5α was used as a host for DNA manipulation.

### Plasmids.

Plasmid pCS299P (37), a C. glutamicum-E. coli shuttle vector, was used to clone the PCR products. Plasmid pESB30 (37), which is nonreplicative in C. glutamicum, is a vector for gene replacement in C. glutamicum. Plasmids pCfadD1, pCfadD4, pCfadD5, pCfadD15, and pCfadD32 were constructed so that *fadD1* (Cgl0284, NCgl0279), *fadD4* (Cgl1198, NCgl1151), *fadD5* (Cgl0400, NCgl0388), *fadD15* (Cgl2296, NCgl2216), and *fadD32* (Cgl2872, NCgl2774) were constitutively expressed under the control of the promoter of the endogenous *gapA* gene. For the construction of pCfadD15, the open reading frame (ORF) of *fadD15* was amplified using primers fadD15sdFusF and fadD15FusR, with wild-type ATCC 13032 genomic DNA as the template. On the other hand, the genomic region comprising the *gapA* promoter was amplified using primers PgapAKpBgF and PgapAfadD15sdFusR so that the ribosome-binding site (RBS) sequence for *gapA* was altered to the consensus RBS sequence proposed for C. glutamicum (38). Similarly, the genomic region comprising the *gapA* terminator was amplified using primers fadD15TTgapAFusF and TTgapAKpR. These three fragments were fused by PCR, digested with KpnI, and then ligated to KpnI-digested pCS299P to yield pCfadD15.

Plasmids for the other four *fadD* genes were constructed by replacing the *fadD15* ORF on pCfadD15 with the ORF of the corresponding genes, as follows. Plasmid pCfadD15 was linearized by inverse PCR using primers InVer-PgapAsdR and InVer-TTgapAF so as to completely remove only the *fadD15* ORF (for convenience, the resulting linear plasmid is referred to here as fragment A). On the other hand, the ORFs of the target genes were amplified using the primer pairs InFu-fadD1F and InFu-fadD1R for *fadD1*, InFu-fadD4F and InFu-fadD4R for *fadD4*, InFu-fadD5F and InFu-fadD5R for *fadD5*, and InFu-fadD32F and InFu-fadD32R for *fadD32*. The amplified ORFs were individually cloned into fragment A using an In-Fusion HD cloning kit (Clontech Laboratories, Inc., Mountain View, CA, USA) to yield pCfadD1, pCfadD4, pCfadD5, and pCfadD32. For *fadD4* on pCfadD4, the native rare start codon GTG was modified to ATG.

Plasmid pCtesA, which contains the *tesA* gene (Cgl2451, NCgl2365), was constructed as follows. The genomic region comprising *tesA* and its native promoter (from −1 to −123 bp upstream of *tesA*) was amplified using primers tesAup120FBamHI and tesAdown70RBamHI. The resulting fragment was digested with BamHI and then ligated to BamHI-digested pCS299P to yield pCtesA.

The sequences of the primers used in this study are listed in Table 2. All primers were designed based on the genomic sequence of C. glutamicum (BA000036) (39), which is publicly available at http://www.genome.jp/kegg/genes.html.

**TABLE 2:** Sequences of primers used in this study

Primer	Sequence (5′ to 3′)^*a*^	Purpose
fadD15sdFusF	CCTACAATCTTGAAAGGAGGCACAACATGACTTCACCTAATACCCTGCAGG	Expression of *fadD15*
fadD15FusR	GCCCACGTTAGCGATGTGAACTAATCATCGGTTGTAGATGTGGTCGATG	Expression of *fadD15*
PgapAKpBgF	GCGGGTACCAGATCTGAAGATTCCTGATACAAATTCTGTTG	Expression of *fadD15*, *fadD1*, *fadD4*, *fadD5*, or *fadD32*
PgapAfad15sdFusR	CCTGCAGGGTATTAGGTGAAGTCATGTTGTGCCTCCTTTCAAGATTGTAGG	Expression of *fadD15*, *fadD1*, *fadD4*, *fadD5*, or *fadD32*
fadD15TTgapAFusF	CATCGACCACATCTACAACCGATGATTAGTTCACATCGCTAACGTGGGC	Expression of *fadD15*, *fadD1*, *fadD4*, *fadD5*, or *fadD32*
TTgapAKpR	AATGGTACCGATTAAAGACACAAAATAGCCC	Expression of *fadD15*, *fadD1*, *fadD4*, *fadD5*, or *fadD32*
InVer-PgapAsdR	GTTGTGCCTCCTTTCAAGATTGTAGGAAATGCAATGTGTC	Expression of *fadD1*, *fadD4*, *fadD5*, or *fadD32*
InVer-TTgapAF	TTAGTTCACATCGCTAACGTGG	Expression of *fadD1*, *fadD4*, *fadD5*, or *fadD32*
InFu-fadD1F	GAAAGGAGGCACAACATGAAAGTGAACCTCGGAATAGGAAG	Expression of *fadD1*
InFu-fadD1R	AGCGATGTGAACTAATTATGAAACGGGGATGGTGAAGTC	Expression of *fadD1*
InFu-fadD4F	GAAAGGAGGCACAACATGTCGCATGCCCCGGCTATTGTTG	Expression of *fadD4*
InFu-fadD4R	AGCGATGTGAACTAATTATTTTTCACCTGGCCCTTTAAGC	Expression of *fadD4*
InFu-fadD5F	GAAAGGAGGCACAACATGTCAGCATACGAAACCAAAGAATGGC	Expression of *fadD5*
InFu-fadD5R	AGCGATGTGAACTAACTACTTGCCGAGCTTCTTCAACAAC	Expression of *fadD5*
InFu-fadD32F	GAAAGGAGGCACAACATGGATTTAGATAAAGCGATTGGTTCATTC	Expression of *fadD32*
InFu-fadD32R	AGCGATGTGAACTAACTAGTTAGCTTGTTCCTGAATGTAGTTG	Expression of *fadD32*
tesAup120FBamHI	TTG*GGATCC*TTGTCACTAAATGTGCTCAGCTTCG	Expression of *tesA*
tesAdown70RBamHI	AAC*GGATCC*AAACCAACCGCACGCGCAAC	Expression of *tesA*
In-FufadD5up760Fw	TAGAGTCGACCTGCAACTACCTTTGTGTGCAGCGGAATG	Deletion of *fadD5*
fadD5delFusRev	CTTCACGACGCCTAATCTTGCCCATCGAGTGTGGCGTCCACTCTG	Deletion of *fadD5*
fadD5delFusFw	CAGAGTGGACGCCACACTCGATGGGCAAGATTAGGCGTCGTGAAG	Deletion of *fadD5*
In-FufadD5down740Rev	CCAAGCTTGCATGCCCGAAGTCCAAAATCTGCCTGTATGG	Deletion of *fadD5*
P9L	TGCAGGTCGACTCTAGAGGATCCCCGGGTAC	Deletion of *fadD5* and *fadD15*
P10L	GGCATGCAAGCTTGGCGTAATCATGGTCATAG	Deletion of *fadD5* and *fadD15*
In-FufadD15up750Fw	TAGAGTCGACCTGCAATGATCAAACCGCACTCAGCTGAG	Deletion of *fadD15*
fadD15delFusRev	GGTCAGGTCGCGATCAAGGATGTATTCTCCGATGGTGTACTTGGCAGG	Deletion of *fadD15*
fadD15delFusFw	CCTGCCAAGTACACCATCGGAGAATACATCCTTGATCGCGACCTGACC	Deletion of *fadD15*
In-FufadD15down700Rev	CCAAGCTTGCATGCCCGTAGCTAAAGTTCTAGCCGGTCT	Deletion of *fadD15*
tesAup630FBamHI	AGC*GGATCC*CCATGGTTTAGGCCC	Deletion of *tesA*
tesAdelFusR	CTCATCAGTAGCGACAGCGGTCAGGAGATCCACCTCCGTCACGTGAAG	Deletion of *tesA*
tesAdelFusF	CTTCACGTGACGGAGGTGGATCTCCTGACCGCTGTCGCTACTGATGAG	Deletion of *tesA*
tesAdown770RBamHI	CTA*GGATCC*CGTATTCACCCACGG	Deletion of *tesA*
InFu-iolT1up450F	TAGAGTCGACCTGCAGTCCGCTCCTCGCACGCTTTTTGTAA	Replacement of chromosomal *iolT1* with *tesA*
iolT1-tesAFusR	GTCGTTGACATTGTTGGCTGCCATCTTGTCTCCTAAGTTTGTCGTGCC	Replacement of chromosomal *iolT1* with *tesA*
tesA-iolT1downFusF	GTCGCAGGAAGCTCTAGAAAAGTAGAAACCCAGACACTGCATAGATAACACG	Replacement of chromosomal *iolT1* with *tesA*
InFu-iolT1down740R	CCAAGCTTGCATGCCGAAGACTCCACGATCTCGGATATTTC	Replacement of chromosomal *iolT1* with *tesA*
tesAFusForf	GGCACGACAAACTTAGGAGACAAGATGGCAGCCAACAATGTCAACGAC	Replacement of chromosomal *iolT1* with *tesA*
tesAFusRorf	CGTGTTATCTATGCAGTGTCTGGGTTTCTACTTTTCTAGAGCTTCCTGCGAC	Replacement of chromosomal *iolT1* with *tesA*
fadD5_realtime-PCR_F	CTACGCAGTGCTGAAACTCG	qPCR analysis for *fadD5*
fadD5_realtime-PCR_R	TGTCCACGTAGCTGTTCGAC	qPCR analysis for *fadD5*
fadD15_realtime-PCR_F	AGGAGCTGCTGCCACTTCTTC	qPCR analysis for *fadD15*
fadD15_realtime-PCR_R	ATCCCTGGAACAACATCTCG	qPCR analysis for *fadD15*
tesA_realtime-PCR_F	TGAGATCCCTGCTGTGTTTG	qPCR analysis for *tesA*
tesA_realtime-PCR_R	CCGTTACGATCCTTGACCTC	qPCR analysis for *tesA*
rRNA_realtime-PCR_F	CTTACCTGGGCTTGACATGG	qPCR analysis for 16S rRNA
rRNA_realtime-PCR_R	CACCATAATGTGCTGGCAAC	qPCR analysis for 16S rRNA

a KpnI sites are underlined, and BamHI sites are italicized.

### Media.

Complete BY medium and minimal medium (MM) were used as basal media for the growth of C. glutamicum strains (40). Solid plates were made by the addition of Bacto agar (Difco) to 1.5%. For preparation of MM containing sodium oleate or sodium palmitate, the fatty acid sodium salt was separately autoclaved and then mixed with a magnesium sulfate solution and a solution containing other components to prevent insolubilization of the fatty acid. For cultivation of plasmid carriers, kanamycin was added at a final concentration of 10 mg/liter. For growth of E. coli, Luria-Bertani broth or agar was used.

### Recombinant DNA techniques.

Standard protocols (41) were used for the construction, purification, and analysis of plasmid DNA and for the transformation of E. coli. The extraction of C. glutamicum chromosomal DNA and transformation of C. glutamicum by electroporation were carried out as described previously (40). PCR was performed using a DNA thermal cycler (GeneAmp PCR system 9700; Applied Biosystems, Foster City, CA, USA) using Phusion High-Fidelity DNA polymerase (New England Biolabs, Ipswich, MA, USA). Sequencing to confirm the nucleotide sequences of relevant DNA regions was performed using an ABI PRISM 377 DNA sequencer from Applied Biosystems, with an ABI PRISM BigDye Terminator cycle sequencing kit (Applied Biosystems). The subsequent electrophoresis analysis was carried out using Pageset SQC-5ALN 377 (Toyobo, Osaka, Japan).

### Strain construction.

For the chromosomal deletions of *fadD5*, *fadD15*, and *tesA*, plasmids pCΔfadD5, pCΔfadD15, and pCΔtesA, which contained the corresponding genes with internal deletions, were used to replace the wild-type chromosomal genes with the deleted genes. For the construction of pCΔfadD5, the 5′ and 3′ regions of *fadD5* were amplified using the primer pair In-FufadD5up760Fw and fadD5delFusRev and the primer pair fadD5delFusFw and In-FufadD5down740Rev, respectively. These two fragments were fused by PCR and then cloned into linearized pESB30, which was obtained by inverse PCR using primers P9L and P10L, with an In-Fusion HD cloning kit. The resulting plasmid, pCΔfadD5, carried the in-frame-deleted *fadD5* gene, which was shortened from 1,707 to 120 bp. Similarly, for the construction of pCΔfadD15, the 5′ and 3′ regions of *fadD15* were amplified using the primer pair In-FufadD15up750Fw and fadD15delFusRev and the primer pair fadD15delFusFw and In-FufadD15down700Rev, respectively. These two fragments were fused by PCR and then cloned into linearized pESB30, which was obtained by inverse PCR using primers P9L and P10L. The resulting plasmid, pCΔfadD15, carried the in-frame-deleted *fadD15* gene, which was shortened from 1,848 to 180 bp. For the construction of pCΔtesA, the 5′ and 3′ regions of *tesA* were amplified using the primer pair tesAup630FBamHI and tesAdelFusR and the primer pair tesAdelFusF and tesAdown770RBamHI, respectively. These two fragments were fused by PCR, digested with BamHI, and then ligated to BamHI-digested pESB30 to yield pCΔtesA. This plasmid carried the in-frame deleted *tesA* gene, which was shortened from 468 to 117 bp. The defined chromosomal deletion of the individual gene was accomplished using each plasmid via two recombination events as described previously (42).

For the construction of the *myo*-inositol-dependent *tesA*-expressing strains WTtesA^iol^ and WTΔfasRtesA^iol^, the *tesA*-disrupted strains WTΔtesA and WTΔfasRΔtesA, respectively, were used as host strains to replace the chromosomal *iolT1* gene (Cgl0181, NCgl0178), which is expressed under the control of its native *myo*-inositol-inducible promoter (43), with the *tesA* gene. For this gene replacement, plasmid pCPiolT1-tesA was constructed as follows. The upstream and downstream regions of the *iolT1* gene ORF (for convenience, these regions are referred to here as fragments B and C, respectively) were amplified by pairs of primers (InFu-iolT1up450F–iolT1-tesAFusR and tesA-iolT1downFusF–InFu-iolT1down740R, respectively). The *tesA* gene was amplified using primers tesAFusForf and tesAFusRorf. Fragment B, the *tesA* gene, and fragment C were fused stepwise using PCR. The resulting 1.7-kb fragment was cloned using an In-Fusion HD cloning kit into linearized pESB30, which was obtained by inverse PCR using primers P9L and P10L, to yield plasmid pCPiolT1-tesA. The defined chromosomal replacement of *iolT1* with *tesA* in strains WTΔtesA and WTΔfasRΔtesA resulted in strains WTtesA^iol^ and WTΔfasRtesA^iol^, respectively. Although strains WTtesA^iol^ and WTΔfasRtesA^iol^ lack the *iolT1* gene, both strains can use *myo*-inositol as a carbon source due to the existence of an additional transporter encoded by *iolT2* (Cgl3058, NCgl2953).

### Fatty acid production.

A 3-ml sample of the seed culture grown in BY medium to the mid-exponential phase at 30°C was inoculated into a 300-ml baffled Erlenmeyer flask containing 30 ml of MM (1% glucose or 1% *myo*-inositol), followed by cultivation at 30°C using a rotary shaker at 200 rpm. After glucose or *myo*-inositol was consumed, total lipids, including free fatty acids, were extracted from the culture supernatant as described previously (14). In the case of the high-fatty-acid producers PCCA-3 and its derivatives shown in Fig. 6, total lipids were extracted from the culture broth containing cells, because some of the fatty acids produced were likely to be insolubilized in the broth. The extracted lipids were subject to quantitative determination of free fatty acids by gas chromatography as described previously (14).

### Liquid cultures to examine the abilities to utilize oleic acid.

A 0.05-ml sample of the first-seed culture grown in BY medium to the mid-exponential phase was inoculated into 5 ml of biotin-free MM and cultivated for 20 h to deplete biotin in the culture. The resulting second-seed culture was harvested, washed three times with saline, and resuspended in 5 ml of biotin-free MM. The main culture was started by inoculating 0.05 ml of the biotin-depleted second-seed culture into 5 ml of biotin-free MM supplemented with the indicated concentrations of biotin or sodium oleate (Fig. 2). All liquid cultures were performed at 30°C in L-type test tubes on a Monod shaker at 48 strokes/min.

### Growth test of strains WTΔtesA and WTtesA^iol^.

For strain WTΔtesA, a 0.05-ml sample of the seed culture grown in BY medium supplemented with 1% glucose was inoculated into 5 ml of MM (1% glucose) supplemented with the indicated concentrations of sodium oleate or sodium palmitate (Fig. 3A). For strain WTtesA^iol^, a 0.05-ml sample of the seed culture grown in BY medium supplemented with 1% glucose or 0.5% glucose plus 0.5% *myo*-inositol was inoculated into 5 ml of MM containing 1% glucose or 0.5% glucose plus 0.5% *myo*-inositol. All liquid cultures were performed at 30°C in L-type test tubes on a Monod shaker at 48 strokes/min.

### Enzyme assays.

Cells grown at 30°C to the late exponential phase in a 300-ml baffled Erlenmeyer flask containing 30 ml of MM (1% glucose) were collected by centrifugation at 10,000 × *g* for 10 min and washed twice with 50 mM Tris-HCl buffer (pH 8.0) and 150 mM 3-(*N*-morpholino)propanesulfonic acid (MOPS)-NaOH (pH 7.2) for Tes and FadD assays, respectively. The cells were suspended in the corresponding buffer and sonicated on ice for 10 min using a UD-200 ultrasonic disruptor (Tomy Seiko Co., Ltd., Tokyo, Japan). For the Tes assay, cell debris was removed by centrifugation at 10,000 × *g* for 10 min, and the supernatant was further ultracentrifuged at 100,000 × *g* for 90 min using an Optima TL ultracentrifuge (Beckman Coulter, Inc., Fullerton, CA, USA). Using the resulting supernatant, the Tes activity was spectrophotometrically measured at 30°C by the methods described by Barnes (44) with the assay mixture consisting of 50 mM Tris-HCl (pH 8.0), 100 μM 5,5′-dithiobis(2-nitrobenzoic acid), 0.008% bovine serum albumin, 14 μM palmitoyl-CoA, and enzyme solution. One unit of Tes activity was defined as 1 μmol of acyl-CoA cleaved per min.

For the FadD assay, the cell debris after sonication was removed by centrifugation at 10,000 × *g* for 10 min, and the resulting supernatant was used for the FadD assay. The activity was basically measured according to the enzyme-coupled assay described by Ichihara and Shibasaki (45). The assay involved the conversion of substrate (5 mM palmitic acid) to acyl-CoA by the endogenous FadD activity at 30°C in 150 mM MOPS-NaOH (pH 7.2) containing 0.5 mM CoA, 4.5 mM ATP, 12 mM MgCl_2_, 1 mM dithiothreitol, 2 μM FAD, 1% methanol, 0.55 mM Triton X-100, 10 U/ml of acyl-CoA oxidase, and 10,000 U/ml of catalase. Acyl-CoA formed from the fatty acid and CoA by FadD was dehydrogenated by acyl-CoA oxidase and then converted into formaldehyde in the presence of methanol by catalase. The concentration of formaldehyde that originated from acyl-CoA was spectrophotometrically measured at time intervals by the colorimetric method (45). One unit of FadD activity is defined as 1 μmol of acyl-CoA formed per min. Protein content was determined using a Bio-Rad protein assay kit (Bio-Rad Laboratories, Hercules, CA, USA). Data are mean values and standard deviations from triplicate assays.

### RNA extraction, cDNA synthesis, and quantitative PCR.

Extraction of RNAs from C. glutamicum strains and subsequent purification were performed according to the methods described previously (46). cDNA synthesis was performed with 300 ng of RNA using the methods described by Kind et al. (47). Quantitative PCR (qPCR) analysis was performed using the method described by Katayama et al. (48). The gene expression levels were standardized to the constitutive level of 16S rRNA expression and calculated by the comparative cycle threshold method (49).

### Analysis.

Bacterial growth was monitored by measuring the optical density at 660 nm (OD_660_) of the culture broth using a Miniphoto 518 R spectrophotometer (Taitec, Saitama, Japan). Concentrations of glucose, *myo*-inositol, and protein were determined using their respective assay kits, as described previously (50).

## Supplementary Material

Supplemental file 1
