# Supplementary material for: A Futile Metabolic Cycle of Fatty Acyl Coenzyme A (Acyl-CoA) Hydrolysis and Resynthesis in Corynebacterium glutamicum and Its Disruption Leading to Fatty Acid Production
Source: Appl Environ Microbiol. 2021 Jan 29;87(4):e02469-20. doi: 10.1128/AEM.02469-20 (PMC7851686; doi:10.1128/AEM.02469-20)
Supplement: Supplemental file 1 [file AEM.02469-20-s0001.pdf]

SUPPLEMENTAL MATERIAL

A Futile Metabolic Cycle of Fatty Acyl-CoA Hydrolysis and Resynthesis in *Corynebacterium glutamicum* and Its Disruption Leading to Fatty Acid Production

Masato Ikeda[a,*], Keisuke Takahashi[a], Tatsunori Ohtake[a], Ryosuke Imoto[a], Haruka Kawakami[a], Ryosuke Kato[a], Mikiro Hayashi[b], Seiki Takeno[a]

Department of Agricultural and Life Sciences, Faculty of Agriculture

Shinshu University, Nagano 399-4598, Japan[a]

Bioprocess Development Center, Kyowa Hakko Bio Co., Ltd.,

Tsukuba, Ibaraki 305-0841, Japan[b]

***Corresponding author:**

Masato Ikeda

Department of Agricultural and Life Sciences, Faculty of Agriculture, Shinshu University, Nagano 399-4598, Japan

Phone: +81-265-77-1614; Fax: +81-265-77-1629; E-mail: m_ikeda@shinshu-u.ac.jp

A

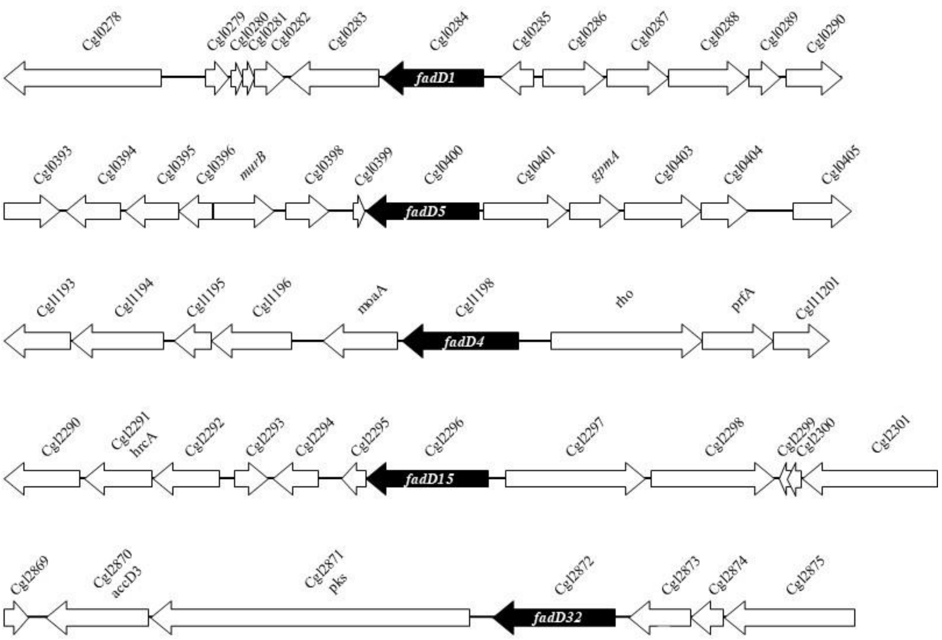

B

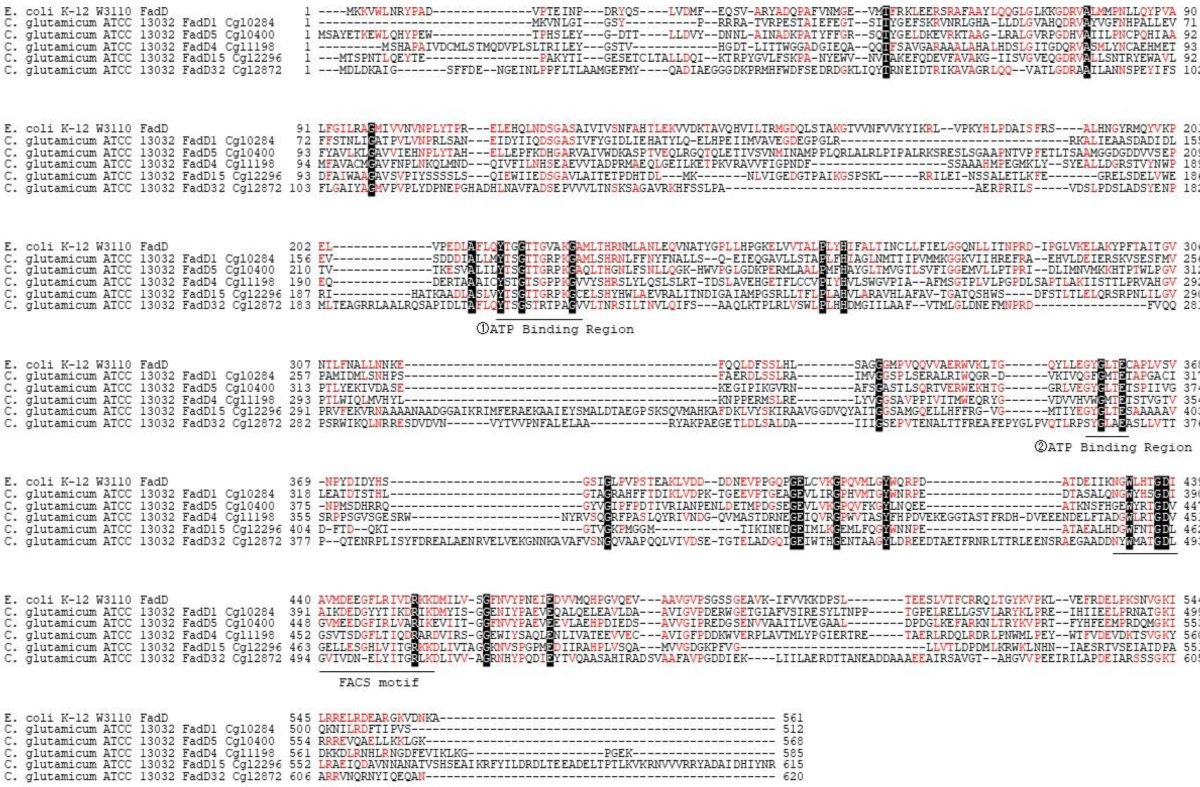

FIG S1 Structural organization of the putative *fadD* genes of *C. glutamicum* (A) and alignment of the gene products with the *E. coli fadD* gene (B). (A) The arrows indicate the relative size and transcriptional direction of the genes. The numbers and names above the arrows indicate the gene annotations. (B) Conserved amino acids are indicated in red. Shaded amino acids in white are conserved among all *fadD* sequences. ATP-binding and fatty acyl-CoA synthetase (FACS) motifs are indicated by underlining.

A

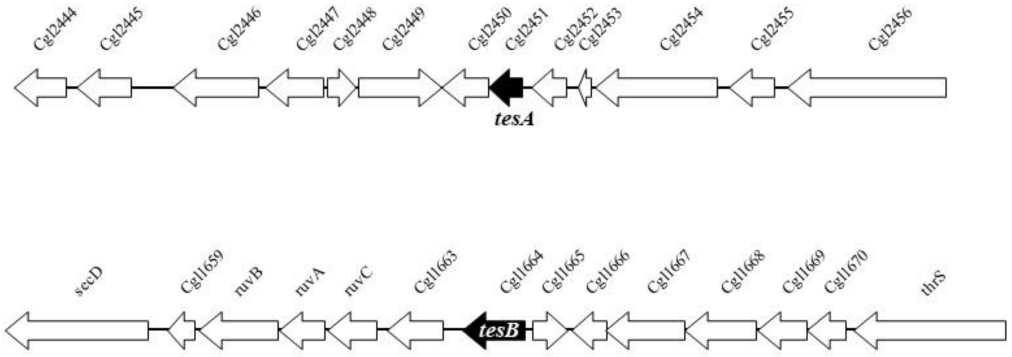

B

```
E. coli K-12 W3110 ybaW          1 --------------MQTQIKVRGYHLDVYQHVNNARYLEFLEEARWDGLENSDSFQWMT-AHNIAFVVVNININYRRPAVLSDLLTI-TSQLQQLNGKSGILSQVITLEPEGQVVADALI 104
C. glutamicum ATCC 13032 tesA     1 MAANNVNDDTQNNLHVTEVDLRWADFDRFGHVNNAAFIEIAQEARLAFAEDQ--FRERG-YEIPAVFVRHLEVDYLRAILPDTTQAVVETQVTKI-GNTSFSTRQEVKDRNGRVCC---- 112
C. glutamicum ATCC 13032 tesB     1 ------MKTIEDIL-----TLEEIDRDIYRGPVIESYLAR----TFGGQVAAQALVAATHTVDKAFTVHSLHGYFIAPGDPTAPA---IYLVDRVRDGKSYVTRSVRGIQDGEVIFSMQA 102
                                                     Active-site motif

E. coli K-12 W3110 ybaW        105 TFVCIDL-----KTQKAL----ALEGE--------------LREKLEQMVK--------------------------------------------------------------------- 132
C. glutamicum ATCC 13032 tesA   113 VVECVQV-----AVNVQTAAPRSISKVERKVLTAVATDEVQSQEALEK------------------------------------------------------------------------ 155
C. glutamicum ATCC 13032 tesB   103 SFHRGDEGIEHMDKMRKVPAPDEIKGTVER-------MPISSRRVLDEWAEWDIRVIPQDQLELSDFTATEQAVWIRCTADLPDNPTFHQCSLTYLSDMTLLHSALVPHPGEKMQMASLD 215

E. coli K-12 W3110 ybaW        132 ------------------------------------------------------------------- 132
C. glutamicum ATCC 13032 tesA   155 ------------------------------------------------------------------- 155
C. glutamicum ATCC 13032 tesB   216 HAVWFLRPFRVDEWLLYDQRSPSASSGRALTHGRLFNQQGDLVAIVNQEGMTRTLHEGAQSIPMRKD 282
```

FIG S2 Structural organization of the putative *tes* genes of *C. glutamicum* (A) and alignment of the gene products with the *E. coli ybaW* gene encoding Tes III, a long-chain acyl-CoA thioesterase (B). (A) The arrows indicate the relative size and transcriptional direction of the genes. The numbers and names above the arrows indicate the gene annotations. (B) Conserved amino acids are indicated in red. Shaded amino acids in white are conserved among all *tes* sequences. The active-site motif homologous to the *E. coli ybaW* product is indicated by underlining.

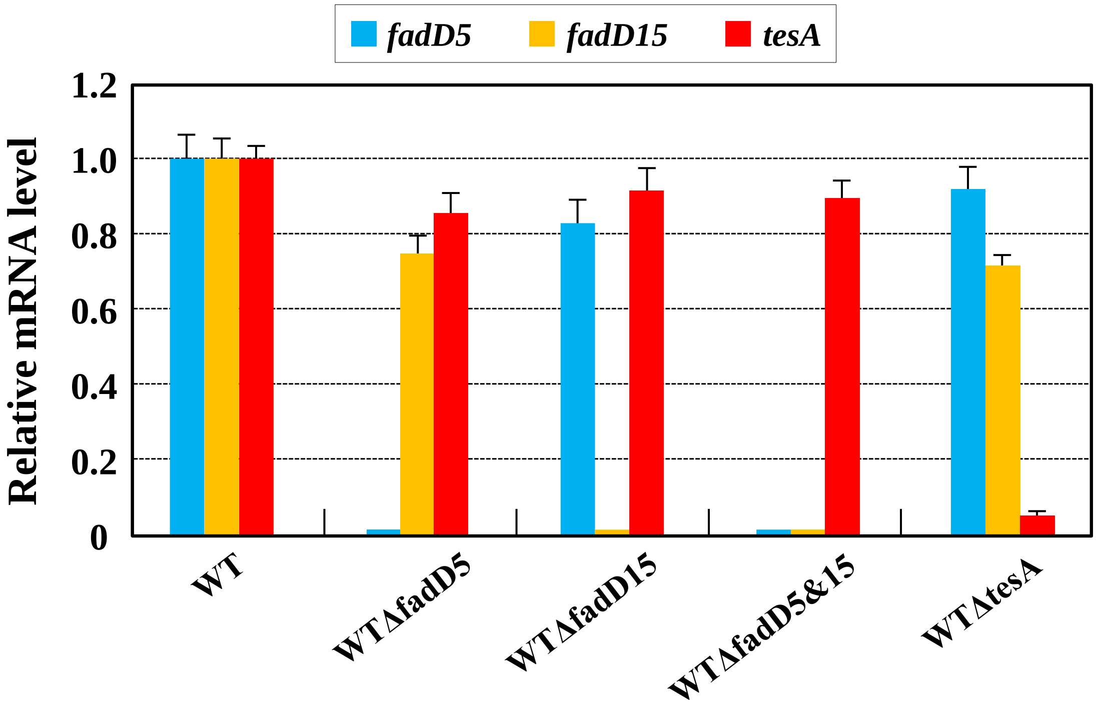

FIG S3 Relative mRNA levels of *fadD5*, *fadD15*, and *tesA* during growth on glucose. Total RNAs were prepared from cells grown to the mid-exponential phase of growth in MM medium. Aliquots of RNAs were reverse-transcribed and then subjected to quantitative PCR. The transcript levels of *fadD5*, *fadD15*, and *tesA* were standardized to the constitutive expression level of 16S rRNA. The transcript levels in the wild-type strain WT were set to 1.0. Data represent means and standard deviations of the results from three independent experiments.
